# The role of surface tension on the growth of bubbles by rectified diffusion

**DOI:** 10.1016/j.ultsonch.2023.106473

**Published:** 2023-06-10

**Authors:** W.R. Smith, Q.X. Wang

**Affiliations:** a School of Mathematics, University of Birmingham, Edgbaston, Birmingham B15 2TT, UK

**Keywords:** Rectified diffusion, Numerical simulation, Aqueous surfactant solutions, Surface tension, Sensitivity

## Abstract

Rectified diffusion has wide and important applications in sonochemistry, ultrasonic cleaning and medical ultrasound. Recent experimental results have demonstrated that the addition of surfactant substantially enhances bubble growth rate. As a hypothesis, this was widely attributed to the acoustic microstreaming and mass transfer resistance caused by surfactants. In this research, the effects of the surfactant of sodium dodecyl sulphate on the rectification have been simulated by considering only the variation of the surface tension coefficient due to the surfactant. The computations are carried out using a newly developed tractable model based on the multi-scale method and the method of matched asymptotic expansions, which allows the prediction of bubble growth taking place over millions of oscillation cycles. The rate of bubble growth observed in the experiments is accurately predicted by our computations, for a range for bulk surfactant SDS concentrations less than or equal to 2.4 mM. Contrary to the widely held hypothesis in the published literature, this has demonstrated that the dominant physical mechanisms remain the shell and area effects in this range of bulk surfactant concentrations. The further enhancement of bubble growth rate provided by either acoustic microstreaming or the resistance to mass transfer is only evident at higher bulk surfactant concentrations. Therefore, the role of surface tension in rectified diffusion for aqueous surfactant solutions is more significant than previously understood. The new results also show that the bubble growth rate is sensitive to small changes in the bubble radius which may account for its unpredictability in applications of sonochemistry.

## Highlights

• The rate of spherical bubble growth is predicted by the reduction of surface tension alone for a range of bulk surfactant concentrations.
• The role of surface tension in rectified diffusion for aqueous surfactant solutions is more significant than previously understood.
• The bubble growth rate is sensitive to small changes in the bubble radius which may account for its unpredictability in applications of sonochemistry.

## 1 Introduction

Rectified diffusion is the growth of bubbles when subject to an acoustic field. Experiments have shown that surface active species, such as surfactants, enhance the bubble growth rate in rectified diffusion. However, a full understanding of the mechanisms of bubble growth from the nanoscale to the microscale underlying this influence remains elusive in part due to the absence of predictive theoretical models.

Blake [1] discovered that, when a bubble experiences nonlinear oscillations, the mass of gas in the bubble may grow rather than the expected process of dissolution of the gas bubble into the liquid. In this incremental process of bubble growth, slightly more dissolved gas in the liquid diffuses across the interface into the bubble than bubble gas diffuses out of the bubble in a process known as rectified diffusion. As almost all liquids contain some dissolved gas in the form of nanobubbles, the application of pressure oscillations may or may not generate bubble growth depending on whether these pressure oscillations have an amplitude above or below a critical threshold value [2], [3].

The underlying mechanisms of rectified diffusion in the bulk have been understood for many decades for air–water systems. The shell and area effects are known to be responsible for the bubble growth [4]. The area effect corresponds to the surface area of the bubble being greater when gas is diffusing into the bubble than when gas is leaving. The shell effect corresponds to the gradient of the concentration of gas in the liquid, or equivalently the mass flux, being greater when the bubble has larger radius. Physical models are well-established [5], [6], [7]. Unfortunately, bubble growth through rectification takes millions of oscillation cycles, the numerical simulations of the process have proved to be very challenging with the convection-enhanced diffusion of gas across the bubble interface being the main difficulty. An analytical expression was found for the gas flux at the bubble interface without neglecting convection [8], [9] which has allowed accurate predictions of the experimental results for rectified diffusion in air–water systems [10], [11], [12], [13].

Acoustic microstreaming corresponds to a flow in the neighbourhood of the bubble which transports fluid with a greater concentration of gas to the bubble interface. Gould [14] has shown that microstreaming can enhance the bubble growth rate, but the enhancement is only significant in the presence of non-spherical surface oscillations for air–water systems. These non-spherical surface oscillations may be activated by a neighbouring bubble or wall. Adjacent to a neighbouring bubble or wall and at resonance, significant microstreaming has been shown to result in convection-dominated bubble growth [15]. Non-spherical surface oscillations may also be generated when the bubble grows to reach its shape mode threshold [4], [16], [17]. In this article, the physical models are for the growth of spherical bubbles due to rectification over millions of oscillation cycles, which are below their shape mode threshold.

Experiments [18], [19], [20] and computations [21], [22], [23] for non-spherical bubbles have been mainly undertaken for a few cycles of oscillation, limited by experimental and computational resources. These studies are far away from the millions of cycles of oscillation that are required for simulating rectified diffusion for non-spherical bubbles.

Initial rectified diffusion experiments in the presence of surfactants were conducted by Crum [11]. The presence of surfactant decreased the threshold for bubble growth and enhanced the growth rate of a single bubble in the bulk substantially. Crum [11] proposed the possible explanation that a low concentration of surfactant could generate microstreaming even in the absence of surface oscillations. Bubble dissolution rate has also been found to be enhanced by the introduction of surfactants in the presence of shape oscillations [24]. The well-established physical model for rectified diffusion was extended in order to explain this enhancement in bubble growth [25]. They incorporated the effect of interfacial resistance to mass transfer caused by the surface materials at the bubble interface. Interfacial resistance to mass transfer is higher when the bubble is smaller as a consequence of the higher surface concentration of surfactant molecules. This inhibits gas flow out of the bubble during the compression phase. It was concluded that interfacial resistance is the fundamental reason for major increases in the bubble growth rates associated with the addition of surfactants [25].

The growth of a single bubble was measured in solutions of the surfactants sodium dodecyl sulphate (SDS) and sodium dodecyl benzenesulfonate [13]. They utilized a non-invasive method for measuring the maximum value of the radius that ensured greater accuracy in comparison with the buoyancy method of Crum [11], [12]. Their results confirmed that the presence of surfactants enhanced the bubble growth rate. Moreover, they provided an experimental basis for the validation of theoretical models which will be especially pertinent to our current study.

The influence of acoustic microstreaming, surface oscillations and the resistance to mass transfer on rectified diffusion was investigated in various aqueous surfactant solutions [26], [27]. These authors discovered that the charge of the ionic surfactant headgroup did not influence the growth rate of bubbles at low surface concentration, but the growth rate was substantially affected by type and charge of the surfactant headgroup at higher surface concentrations. Experimental results showed that, when surfactants are utilised, there was an increase in microstreaming with the bulkiest surfactant headgroup resulting in the greatest increase in microstreaming. Furthermore surface mode oscillations caused a dramatic increase in microstreaming. However, acoustic microstreaming alone could not sufficiently account for the enhancement in bubble growth rate, therefore the impact of the resistance to mass transfer was also investigated. Surfactants with longer chain lengths were shown to enhance the bubble growth rate more than shorter chain lengths [27]. Surfactants with longer chain lengths are associated with greater mass transfer resistance during the compression phase and higher bubble growth rate.

A common theme throughout the published experimental and theoretical literature on aqueous surfactant solutions is that the enhanced bubble growth rate exceeds any effect from the variation of the surface tension. In other words, the shell and area effects are insufficient to account for the rate at which bubbles grow when surfactants are introduced into water. However, no accurate quantitative comparison between computations with the well-established physical model and experiment has been possible to prove this widely-held conclusion. Only the most recent research on rectified diffusion in air–water systems has fully accounted for the convection term in the flux of gas at the interface between the bubble and the liquid. Therefore, our purpose is to undertake this comparison and to explore the bubble growth from the consideration of surface tension coefficient alone in the theoretical model.

The tractable version of the well-established physical model is outlined in § 2 for completeness. For the detailed model, readers should refer to our previous articles [8], [9]. In § 3, a study based on the variation of the surface tension is undertaken. The theoretical results for the time history of the maximum bubble radius are compared with the experimental results for different bulk concentrations of SDS and the addition of salt. The large-amplitude bubble oscillations and the growth rate are also investigated. A brief summary is provided and conclusions are drawn in § 4.

## 2 Physical model

### 2.1 The well-established model

The well-established physical model [8], [9] includes the Rayleigh–Plesset equation for the dimensional spherical bubble radius $\overset{‾}{R}$, the convection–diffusion equation for the dimensional concentration of gas in the liquid $\overset{¯}{c}$ and conservation of mass for the dimensional mass of gas contained in the bubble $\overset{¯}{m}$. In the same order, these equations are (1), (3), (4) as follows

(1) $$\overset{‾}{R}\frac{d^{2}\overset{‾}{R}}{d{\overset{¯}{t}}^{2}}+\frac{3}{2}{\left(\frac{d\overset{‾}{R}}{d\overset{¯}{t}}\right)}^{2}=\frac{{\overset{¯}{p}}_{l}}{\rho },$$

where the pressure of the liquid at the bubble surface less the external applied pressure is ${\overset{¯}{p}}_{l}$ [28] given by

(2) $${\overset{¯}{p}}_{l}={\overset{¯}{p}}_{g}-\frac{2\sigma }{\overset{‾}{R}}-({\overset{¯}{p}}_{\infty }-{\overset{¯}{p}}_{v})-\frac{4\mu }{\overset{‾}{R}}\frac{d\overset{‾}{R}}{d\overset{¯}{t}}-{\overset{¯}{p}}_{a}\sin(2\pi {\overset{¯}{\nu }}_{a}\overset{¯}{t}),$$

and ${\overset{¯}{p}}_{g}$ is given by

$${\overset{¯}{p}}_{g}={\overset{¯}{p}}_{g0}{\left(\frac{\overset{¯}{m}}{{\overset{¯}{m}}_{i}}\right)}^{\kappa }{\left(\frac{{\overset{‾}{R}}_{\mathrm{eqi}}}{\overset{‾}{R}}\right)}^{3\kappa },$$

(3) $$\frac{\partial \overset{¯}{c}}{\partial \overset{¯}{t}}+\frac{d\overset{‾}{R}}{d\overset{¯}{t}}{\left(\frac{\overset{‾}{R}}{\overset{¯}{r}}\right)}^{2}\frac{\partial \overset{¯}{c}}{\partial \overset{¯}{r}}=\frac{D}{{\overset{¯}{r}}^{2}}\frac{\partial }{\partial \overset{¯}{r}}\left({\overset{¯}{r}}^{2}\frac{\partial \overset{¯}{c}}{\partial \overset{¯}{r}}\right)\mathrm{for}\overset{¯}{r}>\overset{‾}{R}(\overset{¯}{t}),$$

(4) $$\frac{d\overset{¯}{m}}{d\overset{¯}{t}}=4\pi {\overset{‾}{R}}^{2}D\frac{\partial \overset{¯}{c}}{\partial \overset{¯}{r}}(\overset{‾}{R}(\overset{¯}{t}),\overset{¯}{t}),$$

with appropriate boundary and initial conditions. Furthermore, Henry’s law provides the boundary condition at the bubble interface

(5) $$\overset{¯}{c}(\overset{‾}{R}(\overset{¯}{t}),\overset{¯}{t})=\frac{{\overset{¯}{p}}_{g}}{k_{H}}=\frac{1}{k_{H}}{\overset{¯}{p}}_{g0}{\left(\frac{\overset{¯}{m}}{{\overset{¯}{m}}_{i}}\right)}^{\kappa }{\left(\frac{{\overset{‾}{R}}_{\mathrm{eqi}}}{\overset{‾}{R}}\right)}^{3\kappa }\text{.}$$

In the above equations, the dimensional radial distance from the centre of the bubble is $\overset{¯}{r}$, the dimensional time is $\overset{¯}{t}$, the initial value of the equilibrium bubble radius is ${\overset{‾}{R}}_{\mathrm{eqi}}$, the liquid density is ρ, the hydrostatic pressure of the liquid is ${\overset{¯}{p}}_{\infty }$, the vapour pressure of the liquid is ${\overset{¯}{p}}_{v}$, the initial pressure of the bubble gas is ${\overset{¯}{p}}_{g0}$ at the mass of gas in the bubble ${\overset{¯}{m}}_{i}$ and bubble radius ${\overset{‾}{R}}_{\mathrm{eqi}}$, the polytropic index is κ>1, the surface tension is σ, the liquid viscosity is μ, the amplitude of the applied pressure is ${\overset{¯}{p}}_{a}$, the frequency of the applied pressure is ${\overset{¯}{\nu }}_{a}$, the mass diffusivity is *D*, the initial uniform concentration of the gas in the liquid is ${\overset{‾}{C}}_{i}$ and the Henry’s law constant is $k_{H}$.

The surfactant concentration on the interface varies due to the changes of bubble surface area during each cycle of oscillation. The surface tension is related to the surfactant concentration via the Szykowsky state equation in the form

(6) $$\sigma =\sigma_{0}+\Gamma_{m}\mathrm{RT}\ln\left(\frac{\Gamma_{m}-\Gamma }{\Gamma_{m}}\right),$$

in which the surface tension in the absence of surfactant is $\sigma_{0}$, the maximum surfactant concentration is $\Gamma_{m}$, the universal gas constant is *R* and the temperature is *T*. Using this formula for the experiments in [13] at the bulk SDS concentration of 1.2 mM, the surface tension typically varies between approximately 0.062 Nm^−1^ and 0.065 Nm^−1^ during each oscillation cycle with the value 0.064 Nm^−1^ corresponding to the equilibrium radius. In the physical model, this variation is assumed to average out over each cycle of oscillation.

The introduction of a surfactant into an air–water system also produces a surface dilatational viscosity, which may have a significant impact on, for example, the small amplitude oscillations of a spherical liquid drop [29]. The pressure at the bubble interface (2) should include an additional term as described in Eq. (18) of [30]. Experimentally measured values of the surface dilatational viscosity vary substantially with the differences being attributed to a wide range of factors [31], [32]. In the following, we assume that the surface dilatational viscosity is insignificant unless our comparisons with experiment indicate otherwise.

In principle, the well-established physical model can be solved numerically. Unfortunately, the physical problem requires millions of oscillation cycles and the computations are associated with billions of time steps. This is feasible for the two ordinary Eqs. (1), (4), but to solve the convection–diffusion Eq. (3) is impractical even taking into account the latest super computers. We thus developed a tractable model to be outlined in § 2.2 utilising the multi-scale method and the method of matched asymptotic expansions.

### 2.2 Summary of the simplified mathematical model

We define the characteristic pressure of the liquid Δ and a reference velocity *U* by

$$\Delta ={\overset{¯}{p}}_{\infty }-{\overset{¯}{p}}_{v},U=\sqrt{\frac{\Delta }{\rho }}\text{.}$$

The corresponding dimensionless parameters are the Reynolds number Re=ρUR‾eqi/μ, the Weber number $\mathrm{We}={\overset{‾}{R}}_{\mathrm{eqi}}\Delta /\sigma$, the dimensionless initial pressure of the bubble gases $p_{g0}={\overset{¯}{p}}_{g0}/\Delta$, the dimensionless amplitude (frequency) of the external pressure $p_{a}={\overset{¯}{p}}_{a}/\Delta$ ($\nu_{a}={\overset{¯}{\nu }}_{a}{\overset{‾}{R}}_{\mathrm{eqi}}/U$), the Peclet number $\mathrm{Pe}=U{\overset{‾}{R}}_{\mathrm{eqi}}/D$, the reciprocal of the dimensionless saturation pressure $d=\Delta /k_{H}{\overset{‾}{C}}_{i}$ and $M={\overset{‾}{R}}_{\mathrm{eqi}}^{3}{\overset{‾}{C}}_{i}/{\overset{¯}{m}}_{i}$.

The Peclet number typically has a value which is greater than one hundred thousand. Therefore the well-established physical model may be simplified using the latest techniques in perturbation theory [33]. This asymptotic analysis was undertaken in our previous article [9]. We now summarise the simplified dimensionless mathematical model.

The dimensionless temporal variable t=t^=Ut¯/R‾eqi corresponds to the shortest time scale for the oscillation of the bubble, the intermediate time scale for the transfer of gas at the interface between the bubble and the liquid Λ=t^/Pe1/2 and the longest time scale for the gas diffusing through the liquid (not immediately adjacent to the bubble) λ=t^/Pe. The Lagrangian change of spatial variable *y* for the gas diffusing through the liquid [34] is defined as

$$y=\frac{1}{3}\left[{\left(\frac{\overset{¯}{r}}{{\overset{‾}{R}}_{\mathrm{eqi}}}\right)}^{3}-{\left(\frac{\overset{‾}{R}}{{\overset{‾}{R}}_{\mathrm{eqi}}}\right)}^{3}\right]\text{.}$$

With the most rapid change on the shortest of the three time scales *t*, the radius of the bubble, $R(t,\Lambda ,\lambda )=\overset{‾}{R}/{\overset{‾}{R}}_{\mathrm{eqi}}$, is governed by the dimensionless Rayleigh–Plesset equation

$$R\frac{\partial^{2}R}{\partial t^{2}}+\frac{3}{2}{\left(\frac{\partial R}{\partial t}\right)}^{2}=\frac{p_{g0}m^{\kappa }}{R^{3\kappa }}-\frac{2}{\mathrm{WeR}}-1-\frac{4}{\mathrm{ReR}}\frac{\partial R}{\partial t}-p_{a}\sin(2\pi \nu_{a}t)\text{.}$$

With the most rapid change on the intermediate of the three time scales Λ, the mass of gas in the bubble, $m(\Lambda ,\lambda )=\overset{¯}{m}/{\overset{¯}{m}}_{i}$, satisfies the dimensionless conservation of mass

$$\frac{\partial m}{\partial \Lambda }=4\pi M\langle R^{4}G\rangle ,$$

where the concentration gradient may be evaluated using

$$\begin{array}{cc} G= & \frac{\left[g(\lambda )-f(0,\Lambda ,\lambda )\right]}{\sqrt{\pi }}{\left\{\int_{0}^{t}R{\left(\eta ,\Lambda ,\lambda \right)}^{4}d\eta \right\}}^{-1/2}\\ & -\frac{1}{\sqrt{\pi }}\int_{\zeta =0}^{t}\frac{\partial f}{\partial \zeta }(\zeta ,\Lambda ,\lambda ){\left[\int_{\zeta }^{t}R{\left(\eta ,\Lambda ,\lambda \right)}^{4}d\eta \right]}^{-1/2}d\zeta , \end{array}$$

in which

$$\langle .\rangle =\frac{1}{P}\int_{t}^{t+P}.dt,$$

$$f(t,\Lambda ,\lambda )=-1+{\mathrm{dp}}_{g0}\frac{m^{\kappa }}{R^{3\kappa }},$$

$$\frac{\partial f}{\partial \zeta }=-3\kappa {\mathrm{dp}}_{g0}\frac{m^{\kappa }}{R^{3\kappa +1}}\frac{\partial R}{\partial \zeta }$$

and P(Λ,λ) is the dimensionless period for the oscillation of the bubble. With changes only on the longest of the three time scales λ, the gas concentration dissolved in the liquid (not immediately adjacent to the bubble), $C(y,\lambda )=(\overset{¯}{c}-{\overset{‾}{C}}_{i})/{\overset{‾}{C}}_{i}$, satisfies the dimensionless diffusion equation

$$\frac{\partial C}{\partial \lambda }=\frac{\partial }{\partial y}\left[\langle {\left(R^{3}+3y\right)}^{4/3}\rangle \frac{\partial C}{\partial y}\right],$$

including the match to the liquid adjacent to the bubble C(0,λ)=g(λ) and the condition in the far field

$$\begin{array}{cc} \frac{\partial C}{\partial y}(L,\lambda )= & -\frac{\lambda^{1/2}C(L,\lambda )}{L^{4/3}}\exp\left(-\frac{{\left(3L\right)}^{2/3}}{4\lambda }\right)\times \\ & \;{\left\{\int_{s=L/\lambda^{3/2}}^{\infty }\frac{1}{s^{4/3}}\exp\left(-\frac{3^{2/3}}{4}s^{2/3}\right)ds\right\}}^{-1}\text{.} \end{array}$$

Dimensionless conservation of the gas in the bubble and the surrounding liquid,

$$\int_{y=0}^{\infty }C\;dy=\frac{1-m}{4\pi M},$$

evaluates g(λ) required by the above matching condition. The initial conditions take the form

$$m(0,0)=1,C(y,0)=0,\frac{\partial R}{\partial t}(0,0,0)=0,R(0,0,0)=1,$$

for 0<y<L.

The computational model has been fully validated against experiments for air–water systems [11], [13] for both bubble growth and dissolution [9]. Convergence tests have also been performed [9].

## 3 Numerical results

It has been postulated in the literature that, with the addition of surfactants, the rate of bubble growth exceeds the effect produced by the reduction of surface tension coefficient alone in the simplified model [11], [12], [13], [25], [26], [27]. Our first task is to investigate this postulate using our validated numerical method. The time history of the maximum bubble radii has been measured for three different bulk concentrations of SDS [13]. The surface tension was ascertained for these three different aqueous solutions with bulk SDS concentrations of 1.2 mM, 2.4 mM and 3.0 mM to be σ=0.064 Nm^−1^, 0.055 Nm^−1^ and 0.048 Nm^−1^, respectively [13]. Therefore, changing these surface tensions alone, three numerical simulations were undertaken in order to draw a comparison with experiment. We emphasize that no other parameters were modified from the comparison with the air–water system in Fig. 3 of [9]. Other parameter values for this system are Re≈240,pg0≈1.1,pa≈0.22,νa≈0.053,Pe≈1.2×105,d≈0.99,M≈9.2×10-3 and κ=1.4 [9].

**Fig. 1:**
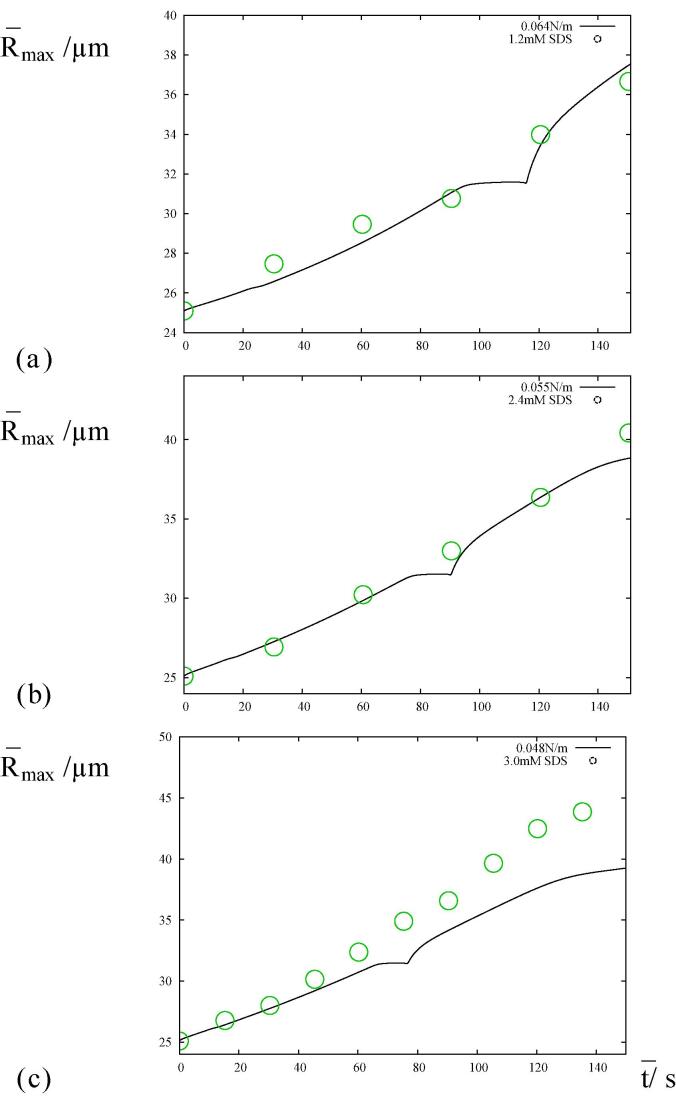
The evolution of the maximum radius which compares three simulations with the experimental results [13]. The experimental set-up comprises a bubble growing in aqueous surfactant solution with the three bulk SDS concentrations of 1.2 mM for (*a*), 2.4 mM for (*b*) and 3.0 mM for (*c*). The corresponding values of surface tension in the numerical simulations are σ=0.064 Nm^−1^ (We≈37) for (*a*), σ=0.055 Nm^−1^ (We≈43) for (*b*) and σ=0.048 Nm^−1^ (We≈49.) for (*c*).

The simulation runs for 150s which encompasses approximately three million bubble oscillations. The theoretical results are shown alongside the experimental results in Fig. 1. Fig. 1, Fig. 1(b) for σ=0.064 Nm^−1^ and 0.055 Nm^−1^ demonstrate that the effect of surface tension alone provides an excellent prediction of the bubble growth, whereas Fig. 1(c) for σ=0.048 Nm^−1^ supports the postulate. At low bulk concentrations, the surface loading of surfactant molecules is shown to be insufficient either to increase the acoustic microstreaming or to decrease the mass transfer during the compression phase of the bubble oscillations. Therefore, at low bulk concentrations, the physics may still be captured by the surface tension coefficient alone.

Furthermore, we can conjecture that there exists a threshold in the bulk surfactant concentration. Below the threshold, the acoustic microstreaming and the resistance to mass transfer have not been activated. Above the threshold, the surface concentration of surfactant has attained a level such that at least one of either microstreaming or mass transfer resistance has been activated. Particle-image velocimetry was used to study the flow surrounding a bubble in water and aqueous surfactant solutions [27]. The mean velocity was measured in order to better understand microstreaming. A 70% increase in mean velocity was found at 3.0 mM SDS in comparison with water which clearly indicates the action of acoustic microstreaming.

**Fig. 2:**
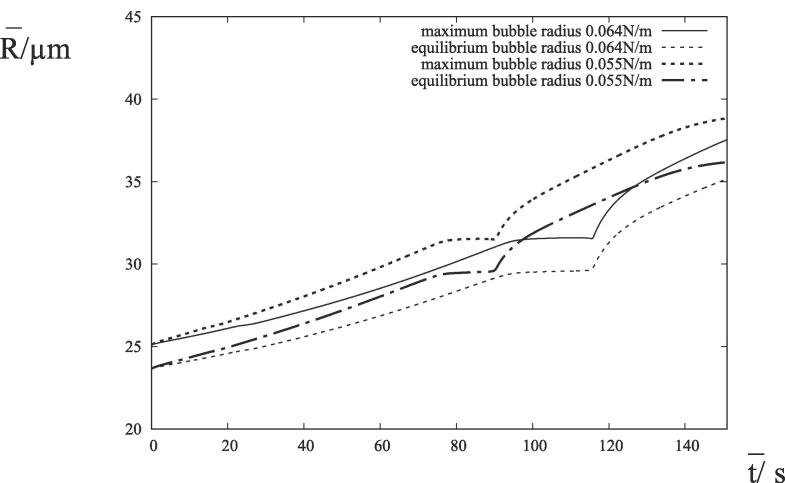
The time histories of the bubble radii with σ=0.064 Nm^−1^ (We≈37) and σ=0.055 Nm^−1^ (We≈43) corresponding to Figs. 1*a*) and 1(*b*), respectively.

An oscillation was seen in the maximum radius at bubble resonance in the absence of surfactant [9] (taking place over many cycles of oscillation) but not in the equilibrium radius. The reduction in surface tension associated with the introduction of surfactant in Fig. 1 has suppressed this oscillation in the dynamics at resonance. The maximum and equilibrium bubble radii follow a similar trend in Fig. 2 and an increase of the maximum radius of bubble oscillation always corresponds to an increase in the equilibrium radius.

For the oscillations of a spherical bubble, the natural frequency is usually stated as follows [3]

(7) $${\overset{¯}{f}}_{0}=\frac{1}{2\pi \rho {\overset{‾}{R}}_{\mathrm{eq}}}\sqrt{\rho \left[3\kappa ({\overset{¯}{p}}_{\infty }-{\overset{¯}{p}}_{v})+\frac{2(3\kappa -1)\sigma }{{\overset{‾}{R}}_{\mathrm{eq}}}\right]-\frac{4\mu^{2}}{{\overset{‾}{R}}_{\mathrm{eq}}^{2}}}\text{.}$$

Using (7), the natural frequency is given by 112 kHz for both ${\overset{‾}{R}}_{\mathrm{eq}}=29.6$ microns and ${\overset{‾}{R}}_{\mathrm{eq}}=29.5$ microns in Fig. 2. The resonance frequency may be seen to be larger than the applied acoustic frequency by a factor of five for the two surface tensions in Fig. 2. Furthermore, Fig. 1, Fig. 2 show that the changes to the bubble growth rate during bubble resonance diminish with a decrease in surface tension.

**Fig. 3:**
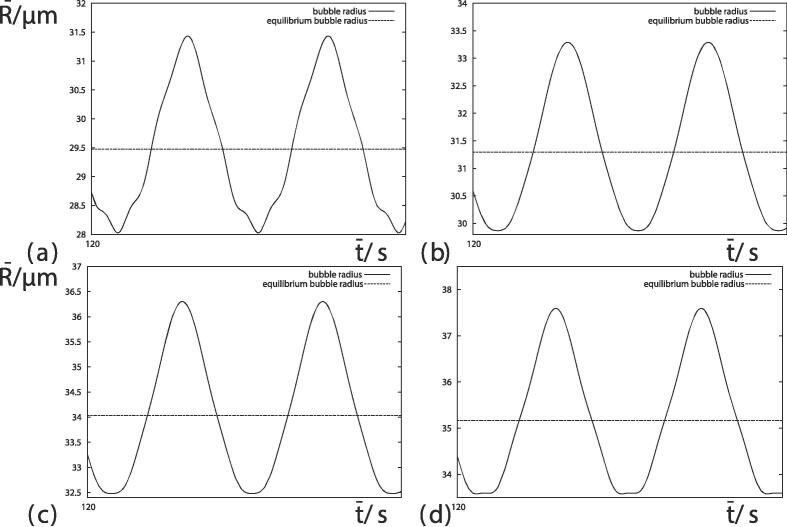
Short histories of $10^{-4}$ s of the bubble radii starting at t=120 s for (*a*) σ=0.072 Nm^−1^ (We≈33), (*b*) σ=0.064 Nm^−1^ (We≈37), (*c*) σ=0.055 Nm^−1^ (We≈43) and (*d*) σ=0.048 Nm^−1^ (We≈49). Other parameters are provided in Fig. 1.

We next analyze the transient oscillations over a few oscillations. Fig. 3 illustrates four histories corresponding to four different values of the surface tension at time t=120 s. The oscillation displays clear nonlinear features, with the maximum bubble radius being above the equilibrium bubble radius by more than the minimum bubble radius is below the equilibrium bubble radius. The greater bubble growth rate at smaller values of the surface tension has resulted in an increasing equilibrium bubble radius and amplitude of oscillation. Fig. 3(a) shows a large-amplitude mode of high frequency corresponding to resonance. Fig. 3, Fig. 3, Fig. 3(d) show that the minima flatten whilst the maxima remain sharp for smaller values of the surface tension. The flattening of the minima would strengthen the shell and area effects.

**Fig. 4:**
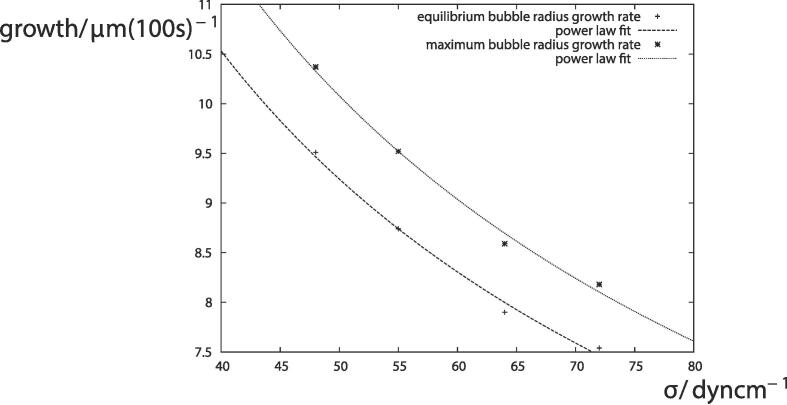
Bubble growth rate as a function of surface tension. The crosses are calculated from numerical simulations of the equilibrium bubble radius and maximum bubble radius when ${\overset{‾}{R}}_{\max}=30\;\mu \text{m}$. Other parameters are provided in Fig. 1.

**Fig. 5:**
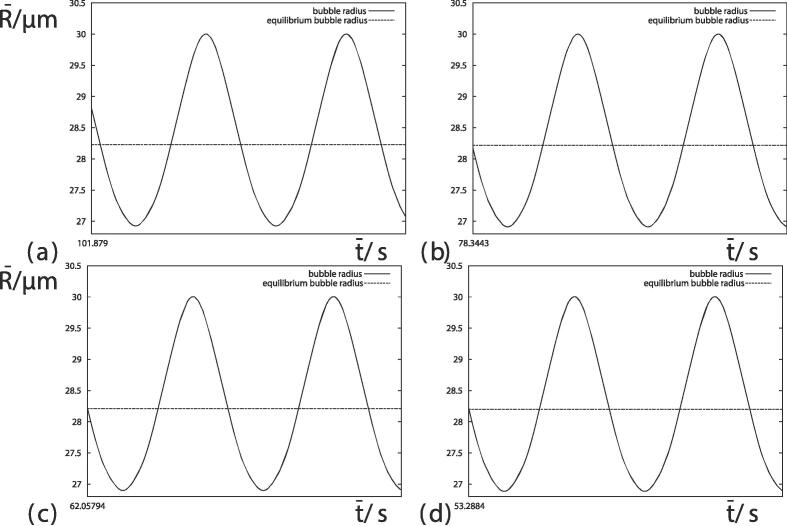
Short histories of $10^{-4}$ s of the bubble radii when ${\overset{‾}{R}}_{\max}=30\;\mu \text{m}$ for (*a*) σ=0.072 Nm^−1^ (We≈33), (*b*) σ=0.064 Nm^−1^ (We≈37), (*c*) σ=0.055 Nm^−1^ (We≈43) and (*d*) σ=0.048 Nm^−1^ (We≈49). Other parameters are provided in Fig. 1.

In order to isolate the effects of surface tension, Fig. 4 shows the bubble growth rate at ${\overset{‾}{R}}_{\max}=30\mu$m as a function of the surface tension. Both the maximum and equilibrium bubble growth rate may be approximated as proportional to the surface tension raised to the power -0.6. Reduction of the surface tension has a significant effect on the growth rate. Fig. 5 compares the time histories of the bubble radius $\overset{‾}{R}$ for a short time period of $10^{-4}$ s for σ=0.072 Nm^−1^, 0.064 Nm^−1^, 0.055 Nm^−1^ and 0.048 Nm^−1^, when ${\overset{‾}{R}}_{\max}=30\mu$m. Apart from a phase shift in Fig. 5(a), the bubble radius appears to be identical for the different values of the surface tension. Closer inspection is required to explain the difference in growth rates. Table 1 provides values of the minimum bubble radius ${\overset{‾}{R}}_{\min}$, the equilibrium bubble radius ${\overset{‾}{R}}_{\mathrm{eq}}$ and the maximum bubble radius ${\overset{‾}{R}}_{\max}$ in Fig. 5 to five significant figures. There are very small differences in the bubble radius. These differences accumulate over millions of oscillations which accounts for the difference in growth rates.

**Table 1:** Numerical values of the minimum bubble radius ${\overset{‾}{R}}_{\min}$, the equilibrium bubble radius ${\overset{‾}{R}}_{\mathrm{eq}}$ and the maximum bubble radius ${\overset{‾}{R}}_{\max}$ shown in Fig. 5 for the four different values of the surface tension σ. Other parameters are provided in Fig. 1.

σ/Nm^−1^	${\overset{‾}{R}}_{\min}$/μm	${\overset{‾}{R}}_{\mathrm{eq}}$/μm	${\overset{‾}{R}}_{\max}$/μm
0.072	26.925	28.227	30.001
0.064	26.911	28.217	30.001
0.055	26.897	28.210	30.004
0.048	26.884	28.201	30.004

**Fig. 6:**
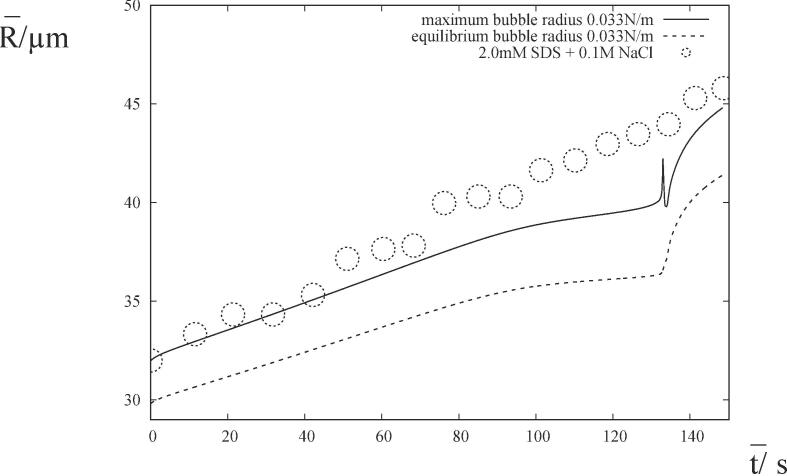
The evolution of the maximum radius which compares a simulation with the experimental results [26] and also the equilibrium radius. The experimental set-up comprises a bubble growing in aqueous surfactant solution with the bulk SDS concentration of 2.0 mM and the addition of 0.1M of NaCl. The corresponding value of surface tension in the numerical simulation is σ=0.033 Nm^−1^ (W.e≈90).

The reduction of surface tension and changes to the electrostatic properties of the bubble are two consequences of the adsorption of SDS at the bubble surface. The addition of 0.1M NaCl neutralizes electrostatic properties as the salt shields the negatively charged headgroup [26]. The removal of electrostatic repulsion allows more surfactant molecules to fit onto the bubble interface which further reduces the surface tension as described in Eq. (6). Little difference was found in the growth rate as a function of the equilibrium surface excess concentration for SDS with and without salt [26]. They concluded that rectified diffusion should be related to surface concentration and not electrostatic properties at low surface loadings. In order to investigate these observations, we consider the experiments in Fig. 2(c) of [26]. The following parameter values for air bubbles in 2.0 mM SDS are taken to be $\Delta =10^{5}$ kgm^−1^s^−2^, σ=0.058 Nm^−1^, κ=1.4,μ=0.001 Pas, $\rho =10^{3}$ kgm^−3^, ${\overset{¯}{\nu }}_{a}=22.31\times 10^{3}$ Hz, ${\overset{¯}{p}}_{a}=2.5\times 10^{4}$ kgm^−1^s^−2^, $D=2\times 10^{-9}$ m^2^s^−1^, $k_{H}=2.5\times 10^{6}$ m^2^s^−2^ and the initial volume fraction of air in the bubble $\phi_{\mathrm{air}}=0.8$. By simulating the experimental results for 2.0 mM SDS in Fig. 2(c) of [26], we deduce the value of the saturation concentration of the gas in the liquid, ${\overset{‾}{C}}_{\mathrm{sat}}=3.94\times 10^{-2}$ kgm^−3^. The initial condition for the concentration of gas in the liquid is then given by ${\overset{‾}{C}}_{i}={\overset{‾}{C}}_{\mathrm{sat}}$. The appropriate value of the surface tension was ascertained to be σ=0.033 Nm^−1^ from Fig. 1 of [35]. (We note that 0.1M NaCl is sufficient to overcome any repulsion caused by electrostatic effects [26], so it will correspond to the surface tension value for 0.15M NaCl in [35].) With all the necessary parameter values now known, the experimental results for 2.0 mM SDS with additional 0.1M NaCl in Fig. 2(c) of [26] are simulated by changing the value of surface tension coefficient alone. Fig. 6 compares the theoretical and experimental results for the maximum bubble radius. Microstreaming and/or interfacial resistance are playing a non-trivial role. The addition of 0.1M NaCl has lowered the bulk concentration of SDS at which microstreaming and interfacial resistance may be neglected. We note that the previous interpretation of the addition of salt was an increase in mass transfer resistance rather than microstreaming [26].

The natural frequency is given by approximately 91 kHz for ${\overset{‾}{R}}_{\mathrm{eq}}=35.8$ microns in Fig. 6. The resonance frequency is four times the applied acoustic frequency. This resonance occurs at later times than observed in the experimental results and corresponds to greater changes in the bubble radius than in Fig. 1, Fig. 2. As described in Appendix C of [7], the polytropic model for the partial pressure of gas in the bubble is responsible for an inaccuracy of the physical model during resonance. There is also an oscillation in the maximum bubble radius, but the equilibrium bubble radius (also shown) does not have such an oscillation. It is a property of the bubble oscillations at resonance and not the underlying bubble growth. This oscillation in the maximum bubble radius at resonance is shown in the experimental results of [13], but it is missing from Fig. 2(c) of [26].

## 4 Summary and conclusions

Rectified diffusion in aqueous solutions of the surfactant SDS has been simulated by the variation of the surface tension coefficient alone in the model. Accurate quantitative comparison between these theoretical results and experimental results has been possible for the first time. The following new phenomena/features are observed:

• Contrary to the widely-held view in the published literature, the rate of bubble growth is predicted by the reduction of surface tension for bulk surfactant SDS concentrations less than or equal to 2.4 mM. The well-established physical model may be utilized to derive accurate quantitative predictions for rectified diffusion in this range of bulk surfactant concentrations.
• Shell and area effects have been identified as the dominant physical mechanisms for rectified diffusion for bulk surfactant SDS concentrations less than or equal to 2.4 mM. The addition of salt may reduce the maximum bulk surfactant SDS concentration at which shell and area effects are the dominant mechanisms.
• The further enhancement of bubble growth rate provided by either acoustic microstreaming or the resistance to mass transfer are only evident at higher bulk surfactant concentrations of SDS.

It has been shown that the bubble growth rate may be greater than SDS for other surfactants (such as dodecyl dimethyl ammonium chloride and dodecyl dimethyl ammonium propane sulphonate) at the same values of the surface tension [27]. This result would indicate that at least one of either acoustic microstreaming or mass transfer resistance is initiated at lower concentrations for these surfactants. However, the role of surface tension should be expected to be greater than previously thought for these other surfactants as well.

The reduction of the surface tension corresponds to significant enhancement of the bubble growth rate, but only very small changes to the dynamics of the bubble. These small changes accumulate over millions of oscillations which accounts for the difference in growth rates. The accuracy required to simulate the bubble radius helps to explain why theoretical methods have proved inadequate over many decades. This sensitivity to small changes in the bubble radius may also go some way to explain the unpredictability in the bubble growth rate in applications of sonochemistry.

## Declaration of Competing Interest

The authors declare that they have no known competing financial interests or personal relationships that could have appeared to influence the work reported in this paper.
